# Therapeutic Radiometals: Worldwide Scientific Literature Trend Analysis (2008–2018)

**DOI:** 10.3390/molecules24030640

**Published:** 2019-02-12

**Authors:** Licia Uccelli, Petra Martini, Corrado Cittanti, Aldo Carnevale, Loretta Missiroli, Melchiore Giganti, Mirco Bartolomei, Alessandra Boschi

**Affiliations:** 1 Department of Morphology, Surgery and Experimental Medicine, University of Ferrara, Via Ludovico Ariosto, 35-44121 Ferrara, Italy; licia.uccelli@unife.it (L.U.); corrado.cittanti@unife.it (C.C.); aldo.carnevale@unife.it (A.C.); melchiore.giganti@unife.it (M.G.); alessandra.boschi@unife.it (A.B.); 2 Nuclear Medicine Unit, University Hospital, Via Aldo Moro, 8-44124 Ferrara, Italy; m.bartolomei@ospfe.it; 3 Legnaro National Laboratories, Italian National Institute for Nuclear Physics (LNL-INFN), Viale dell’Università, 2, 35020 Legnaro (PD), Italy; 4 Radiology University Unit, University Hospital, Via Aldo Moro, 8-44124 Ferrara, Italy; 5 Bibliometric and Databases Unit, Research Office, University of Ferrara, Via Ludovico Ariosto, 35-44121 Ferrara, Italy; loretta.missiroli@unife.it

**Keywords:** therapeutic radiometals, therapeutic radiopharmaceuticals, radionuclide therapy

## Abstract

Academic journals have published a large number of papers in the therapeutic nuclear medicine (NM) research field in the last 10 years. Despite this, a literature analysis has never before been made to point out the research interest in therapeutic radionuclides (RNs). For this reason, the present study aims specifically to analyze the research output on therapeutic radiometals from 2008 to 2018, with intent to quantify and identify global trends in scientific literature and emphasize the interdisciplinary nature of this research field. The data search targeted conventional (^131^I, ^90^Y, ^177^Lu, ^188^Re, ^186^Re, ^153^Sm, ^89^Sr, ^186^Er) and emergent (^67^Cu, ^47^Sc, ^223^Ra, ^166^Ho, ^161^Tb, ^149^Tb, ^212^Pb/^212^Bi, ^225^Ac, ^213^Bi, ^211^At, ^117m^Sn) RNs. Starting from this time frame, authors have analyzed and interpreted this scientific trend quantitatively first, and qualitatively after.

## 1. Introduction

The success of nuclear medicine (NM) has been intimately linked to the availability of new radionuclides (RNs) and the discovery of new radiopharmaceuticals. The field of radiopharmaceuticals is constantly evolving thanks to the great contributions of specialists coming from different disciplines such as physics, inorganic and organic chemistry, radiochemistry, biochemistry, pharmacology, nuclear medicine, and so on. In particular, the research and applications of radiometals for nuclear therapy have experienced a great increase thanks to the development of radionuclide and radiopharmaceutical production technologies, the availability of innovative synthetic strategies, and tools useful for preclinical studies.

The employment of therapeutic radiometals is regulated by their physical characteristics, such as half-life, radiation emission energy and type (β-, γ, auger, α), availability, and chemical ability to coordinate with ligands. In recent years there has been intense research in the field of production and application of conventional therapeutic radionuclides (such as ^131^I, ^90^Y, ^177^Lu, ^188^Re, ^186^Re, ^153^Sm, ^169^Er, ^89^Sr, etc.) (Table 1) compared with emergent therapeutic radionuclides (such as ^67^Cu, ^47^Sc, ^223^Ra, ^166^Ho, ^161^Tb, ^149^Tb, ^212^Pb, ^212^Bi, ^225^Ac, ^213^Bi, ^211^At, etc.) (Table 2) [1,2,3,4,5,6,7,8,9,10,11,12,13,14,15,16,17,18,19,20,21,22,23,24,25,26,27,28]. The great effort that researchers have put in this particular therapeutic field of nuclear medicine is comparable with the research that has been carried out in the last 10 years on Tc-99m alternative production methods [29,30].

New treatment strategies such as the theranostic personalized approach have given a great impulse to the research in this particular field of therapeutic medicine. This approach is based on the association of β^−^ and γ-radiation, which is advantageous in monitoring radiopharmaceutical distribution and therapeutic effects with scintigraphy methods [27]. There are different suitable combinations of molecular targeting vectors and radionuclides for theranostics use. The first is multiple-element theranostics radiopharmaceuticals (different elements showing similar chemical properties—but with different physical emission properties—that are used with the same bioactive molecule, e.g., ^188^Re with ^99m^Tc, ^68^Ga with ^177^Lu or ^90^Y), where one is used for therapeutic tumor treatment and the other is used for diagnosis and response monitoring. Even better is single-element theranostics radiopharmaceuticals, where a bioactive molecule is targeted by different isotopes of the same element with complementary diagnostic and therapeutic properties (e.g., ^43,44^Sc and ^47^Sc) (Table 3), or with a single radioisotope of a single element with both diagnostic and therapeutic emissions (e.g., ^64^Cu suitable for positron emission tomography, PET, imaging and therapy) (Table 4).

The present study has the aim of specifically analyzing the research output on therapeutic radiometals from 2008 (January) to 2018 (October). In this period, authors have quantitatively first, and qualitatively after, analyzed and interpretated this scientific trend. Two databases were taken into consderation: Web of Science (WoS) and Scopus. WoS has been for a long time the only available resource for large-scale bibliometric data, and still today remains the only choice for the scientific impact evaluation of publications before the mid-1990s. The system core, slowly but constantly expanding, is confined in the WoS Core Collection, which indexes over 12,000 journals, 150,000 conference proceedings, and 60,000 monographs, with another 75,000 already pre-announced. WoS selects and discards sources within certain stringent qualitative and quantitative criteria, guided (and justified) by bibliometric rules. Qualitative parameters, besides the obvious peer review requirement, include publication punctuality, English language (at least for titles, abstract, and keywords sections), author and editorial board cosmopolitanism, and editorial content innovation and adequacy, which are ascertained on the basis of a bibliometric mapping of the existing periodic literature.

Quantitative parameters, on the other hand, include impact factor, total number of citations, self-citation percentage, and authors’ citation score (H index).

Overall, the application of these criteria for the WoS selection confirms its elective affinities with the European and North American periodic literature written in English in the natural and biomedical sciences field.

Despite being a newer database, Scopus (from Elsevier) boasts an impressive coverage: over 20,000 peer reviewed journals, 400 trade publications, 370 book series, and 6 milion conference papers coming from proceedings and journals. Moreover, thanks to its integration with the Scirus search engine, millions of scientific patents and web pages are also retrievable from the Scopus database. Sources are selected based on qualitative and quantitative criteria similar to those used by WoS, but with greater weight on qualitative over quantitative parameters independent of the IF value. The selection is not an exclusive affair of Elsevier’s editorial staff, but avails itself of the advice of an independent Content Selection and Advisory Board (CSAB) made up of scientists and librarians from various geographical and disciplinary backgrounds. On the geographical-linguistic level, Scopus is more ecomenical than WoS. More than half of its content comes from areas other than North America. The European presence is especially massive, and over 20% of sources in languages other than English. From the disciplinary point of view, over 70% of the literature collected comes from the physical and biomedical sciences field [32]. 

Academic journals have published a large number of papers in the therapeutic NM research field in the last 10 years. Despite this, a literature analysis has never been made before to address the research interest and potential of therapeutic radionuclides [33]. For this reason the present study aims to specifically analyze the research output regarding therapeutic radiometals from within the past decade, with intent to quantify and identify the global trend of scientific literature and emphasize the interdisciplinary nature of this research field. 

## 2. Results and Discussion

### 2.1. The Worldwide Output

An analysis of global trends was conduced by comparing the data collected by consulting both Scopus and Web of Science (WOS) databases on 16 October 2018 from 3 to 6pm. 

In Table 5a,b, the total data extracted from both databases are listed. The data represent the total number of articles, articles in press, reviews, notes, and short surveys (generally called “papers” for the sake of simplicity) for each radionuclide over the decade. Finally, the radionuclides are divided in two categories: conventional (CRNs) and emergent (ERNs) radionuclides.

A comparison of the total number of papers extrapolated by Scopus vs. WoS (CRNs: 10339 vs. 8435, ERNs: 2378 vs. 1870, respectively) determined the Scopus database as the better source of data for use in the following numeric analysis of literature. This discrepacy can be explained by the less restrictive selection criteria used by Scopus, which favors qualitative over quantitative parameters without restrictions on the journal IF. The database choice is therefore justified, considering the lack of scientific production extrapolated for some radionuclides in particular, including ^149^Tb, ^211^At, and ^186^Er. 

### 2.2. Annual Trend Evaluation

**Figure 1:**
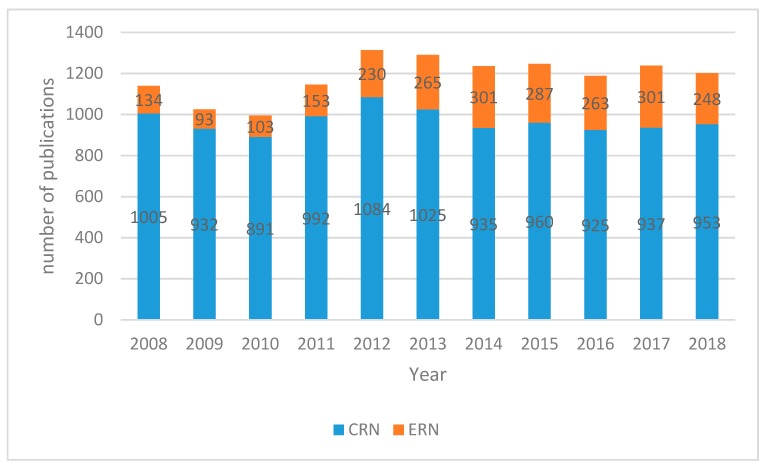
A comparison between the annual publication number of CRNs vs. ERNs (2008–2018). Total number of papers = 12717; total number of papers on CRNs = 10339; total number of papers on ERNs = 2378.

**Figure 2:**
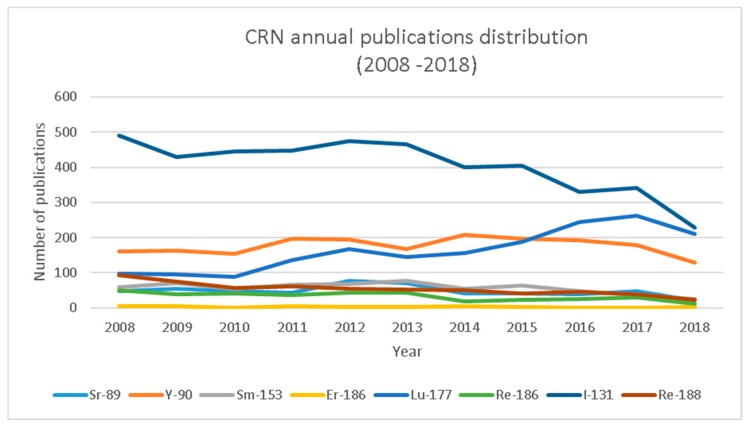
Graphical representation of the annual publications discussing CRNs (2008–2018).

**Figure 3:**
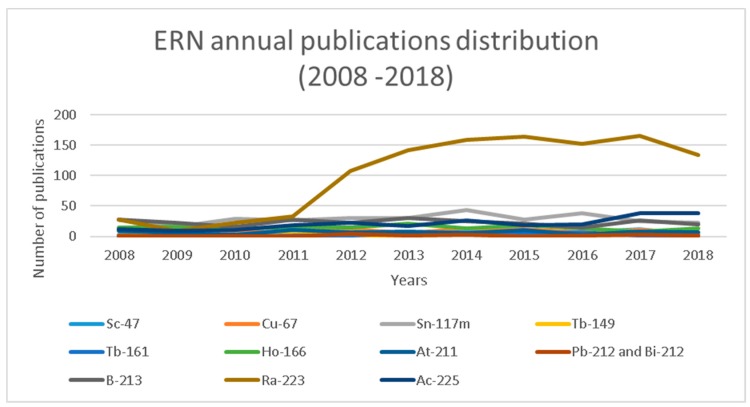
Graphical representation of the annual publications discussing ERNs (2008–2018).

A first analysis of Scopus-collected data of the past 10 years (Figure 1) indicates a consistent annual production of scientific publications discussing conventional therapeutic radionuclides. Since 2012, however, a significant increase of publications concerning emergent radionuclides is noticeable. In order to identify which radionuclides have particularly drawn the scientific community’s attention, Table 6 and Table 7 explicit the annual number of scientific production per radionuclide. Table 6 summarizes the data for conventional radionuclides, and from the data a scientific production stability can be gauged, in particular for Sm-153, Sr-89, and Y-90, with a significative difference in the absolute values for single radionuclides. As regards I-131, there is a significant decline in the number of publications since 2015. This is due to a few different factors: (1) the “2015 American Thyroid Association Management Guidelines for Adult Patients with Thyroid Nodules and Differentiated Thyroid Cancer”, published by the American Thyroid Association Guidelines Task Force on Thyroid Nodules and Differentiated Thyroid Cancer, which has scaled down the use of thyroid ablative treatment; (2) the important prognostic value of PET with ^18^F-FDG, which has limited the use of treatments in patients refractory to iodine; (3) the availability of alternative treatments with biological drugs; and ultimately, (4) the greater interest of researchers towards more innovative therapies. A slight decline characterizes the scientific output related to renium isotopes, which is reasonably matched by the number of Lu-177 publications slowly but constantly growing since 2012 (Figure 2). On the other hand, Table 7 represents the publication distribution for each emergent radionuclide per year. Unsurprisingly, scientific production on Ra-223 substantially rose in the second half of the decade (Figure 3), certainly a result of the marketing authorization of Xofigo (Ra-223 dichloride radiopharmaceutical by Bayer AG) issued by the European Medicines Agency in 2013 [34]. Similarly, in the past two years, scientific literature on Ac-225 has also started experiencing a rise, probably thanks to the increased availability and improved radiochemistry of alpha particle-emitting nuclides for targeted therapy.

### 2.3. Interdisciplinary Nature of Research

**Figure 4:**
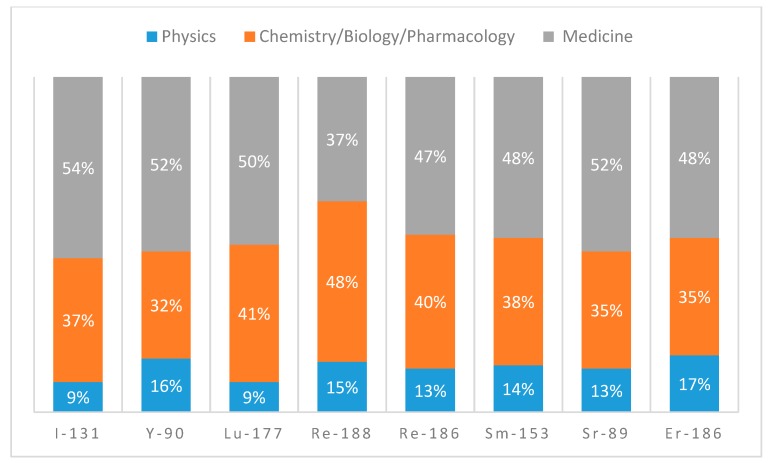
CRN macro-areas contribution (2008–2018).

In order to bring out the multidisciplinary nature of the research in this field, a comparative analysis of the bibliometric data categorized in terms of Journal Subject Areas (JSA) was performed. JSA have been grouped into three large macro-areas (physics, chemistry and biology, and pharmacology and medicine) and divided into each radionuclide. From the analysis of the data relating to conventional radionuclides (Figure 4) that have been widely studied and employed in the last decade, it is clear that researchers who deal with preclinical and clinical application are very (and almost comparably) active. On the other hand, literature regarding the production of conventional radionuclides under the macro-area of physics is poor. Moreover, an in-depth analysis of the publications under this last macro-area indicates that the majority of the publications concern dosimetry studies.

**Figure 5:**
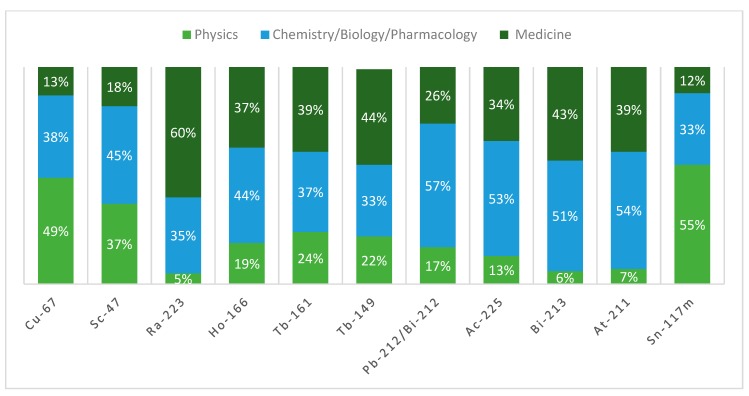
ERN macro-areas contribution (2008–2018).

On the contrary, the data relating to emergent radionuclides (Figure 5) display a significant contribution from areas related to physics, in particular in the case of Cu-67, for which almost 50% of the publications belong to journals of physics, 38% consist of preclinical studies, and only 13% come from studies related to human application. Sc-47 (37% of the 65 publications), and Sn-117m (55% of the 315 publications) are also notable. Regarding Ac-225 and Bi-213, most of the work in the last 10 years has been done on preclinical (Ac-225 = 53% of the 231 publications, Bi-213 = 51% of the 252 publications, At-211 = 54% of the 75 publications) and clinical (Ac-225 = 34% of the 231 publications, Bi-213 = 43% of the 252 publications) studies. Regarding the pair Pb-212 and Bi-212, the majority of the works belongs to the preclinical field (57% of the 14 publications). Finally, and not surprisingly, Ra-223 was dominant in clinical studies, with 60% of works belonging to the medical area.

### 2.4. Country Research Distribution

For each publication, authors’ country of origin was extrapolated and elaborated on to map the global research interest and involvement of human and financial resources. From a preliminary assessment classified by the type of radionuclide, 98 countries study conventional radionuclides while only 85 study emergent radionuclides. Out of a total 98 countries, it turns out that five Asian countries, two countries on the American continents, and six European countries are involved only in research on conventional radionuclides. 

**Figure 6:**
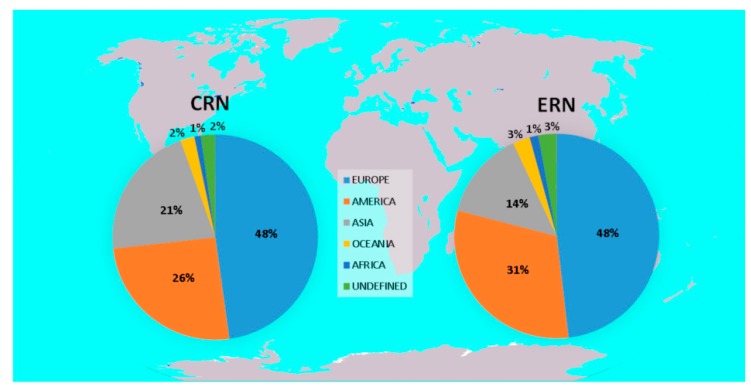
Graphical representation of the worldwide contribution to publications discussing CRNs and ERNs.

In Figure 6, the percentage of publications per continent when compared to the total number of publications produced in the past 10 years is reported for both conventional and emergent radionuclides. This analysis reveals that European researchers are authors or co-authors of almost half of the publications on both conventional and emergent radionuclides. Papers collected without reference to a country were place in the “undefined” category.

**Figure 7:**
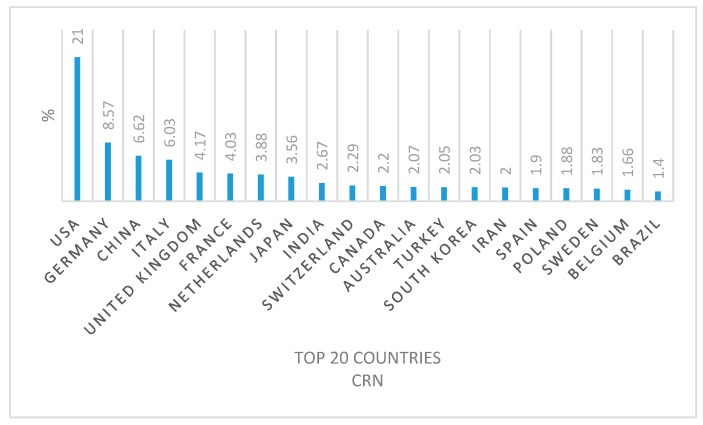
The top 20 countries most contributing to conventional radionuclide publications (2008–2018).

**Figure 8:**
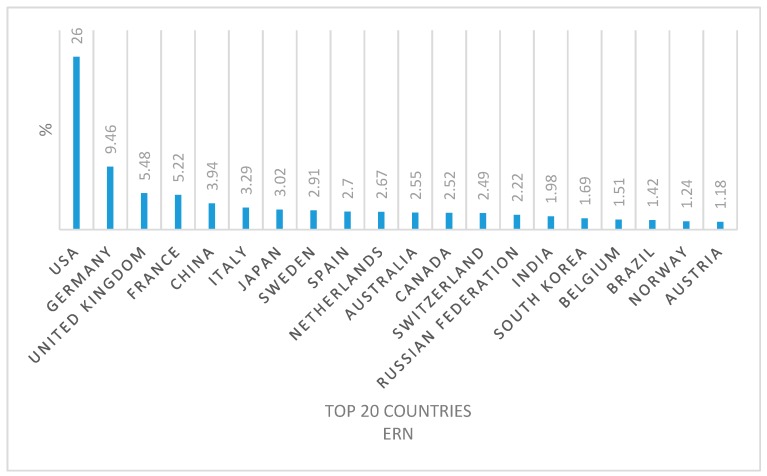
The top 20 countries most contributing to emergent radionuclide publications (2008–2018).

From the list of countries involved in this research field it was also possible to draw up the top 20 countries with the most active researchers (Figure 7 and Figure 8).

At first it is evident that, even if they are in a different order, the countries of the two top 20s are the same. Deepening the analysis however, we notice that USA and Germany rank high in both top 20s, while China slides down from the third position in the conventional radionuclides top 20 to the fifth position regarding emerging radionuclides, thus highlighting a lower investment in human and financial resources. This is similar to Italy, which slides down from fourth to sixth position. On the contrary, the United Kingdom and France both gain two positions from the conventional to emergent radionuclide top 20. 

The percentage of publications, expressed for each conventional and emergent radionuclide, has been calculated for all 98 and 85 countries respectively. However, we in Table 8 and Table 9 the results just for the top-20 countries of the two categories.

In Table 8 and Table 9, however, we show the results only of the the top-20 countries of the two categories.

All countries, at varying rates, studied almost all radionuclides except for Erbium-186. Out of this list we must report Taiwan (rank = 23) that produced 7.24% of the publications on Re-188, giving it fourth place. Similarly, the Czech Republic (rank = 25) tied with USA and Italy by producing 6.52% of the publications on Er-186. 

Out of this list we must report the Czech Republic (rank = 21), which produced 6.02% of the publications on Sc-47, thus gaining the third place tied with Japan. Similarly, Hungary (rank = 26) tied the Russian Federation by producing 11.54% of the publications on Tb-161. 

Denmark (rank = 24) produced 10.91% of the publications on At-111, placing it third.

Analyzing the total amount of affiliations and papers for each category of radionuclide (CRN or ERN) reported in Table 10, it can be deduced that the larger the number of affiliations when compared to the number of publications (12820 over 10339 for CRNs and 3372 over 2378 for ERNs), the higher the grade of cooperation between that countries in this scientific sector.

## 3. Materials and Methods

### 3.1. The Worldwide Output

With the aim to quantify and identify a global trend of scientific literature from the past decade in regards to the interdisciplinary nature of research on therapeutic radionuclides, bibliometric data (number of papers, year of publication, authors’ country of origin) was collected by consulting Scopus (https://www.scopus.com) and Web of Science (WOS) (http://apps.webofknowledge.com) databases on 16 October 2018 from 3 to 6 pm. 

The selection criteria used to refine data collection concerned the following: (1) the search combination terms placed the OR boolean operator among different nomenclature of radionuclides (e.g., ^67^Cu, Cu-67, Copper-67); (2) the search terms were limited to article title, abstract, and keywords; (3) output from the search were further selected by limiting the type of publication via followig filters: article, article in press, note, short survey, review, and letter. Congress abstracts were excluded due to the less-strict peer review selection to which they are subjected. 

The final refined data, reported in Table 5a,b, was finally divided into two classes of radionuclides: coventional (^131^I, ^90^Y, ^177^Lu, ^188^Re, ^186^Re, ^153^Sm, ^89^Sr, ^186^Er) and emergent (^67^Cu, ^47^Sc, ^223^Ra, ^166^Ho, ^161^Tb, ^149^Tb, ^212^Pb/^212^Bi, ^225^Ac, ^213^Bi, ^211^At, ^117m^Sn). 

Scopus was selected as the database of reference for all subsequent analysis, and the list of publications by radionuclide was exported as file.csv (through the function “export refine”). The file was converted to an xls spreadsheet and elaborated upon with Microsoft Office Excel. 

### 3.2. Annual Analysis of Scopus Data Collected

The data in the xls sheet was divided by year of publication and the ∑ (sum) function was applied to calculate the total number of publications per radionuclide per year, as reported in Table 6 and Table 7 and Figure 1, Figure 2 and Figure 3.

### 3.3. Multidisciplinary Nature of Research

In order to highlight the highly multidisciplinary nature of the research of this particular scientific sector and to quantify the contribution to its development given by researchers belonging to various disciplines, the main Journal Subject Area (JSA) was identified for each work. Subsequently, the JSAs were grouped into three large macro-areas, as described in Table 11.

For each category of radionuclides (conventional in Figure 4 and emergent in Figure 5), the percentage of publications belonging to a specific macro-area for each radionuclide was reported.

### 3.4. Countries Research Distribution

For each publication, authors’ country of origin was extrapolated and elaborated upon to map the global research interest and involvement of human and financial resources (each country was counted only once per publication if several authors belonged to the same country independently from the institution). The sum of the contribution percentages from each country on conventional and emergent radionuclides was divided into continents and reported in Figure 6. The top 20 countries that most contributed to research on conventional and emergent radionuclides are reported in Figure 7 and Figure 8. Table 8 and Table 9, on the other hand, analyze the specific contribution percentage of each country per individual radionuclide. 

## 4. Conclusions

A total of 12,717 publications were analyzed, with 81.3% of the publications discussing conventional RNs while 18.7% discussed emergent RNs. The most investigated therapeutic RNs were I-131, Y-90, Lu-177 among conventional, while Ra-223, followed by Sn-117m, Bi-213, and Ac-225, were favored among emergent RNs. From the analysis the multidisciplinary contributions to this field are evident, but in particular, as expected, most publications came from preclinical and clinical fields in the case of conventional RN, while for the emergent RNs the contribution was weighted towards physics, engineering, and materials science fields. From the geographical point of view, we can see how almost half of the total works were published by Europeans both in regards to conventional and emergent RNs. It is also evident that the high collaboration level between countries was characteristically in line with the multidisciplinary character of this medical sectors. Moreover, we extrapolated a ranking of the top 20 countries for each category. In the top three for conventional RNs were USA, Germany, and China, while for emergent RNs it was USA, Germany, and the United Kingdom. 

From this analysis it can be seen that the success of NM has been intimately linked to the availability of new RNs, and that the radiopharmaceuticals field is constantly evolving thanks to the contribution of specialists coming from different disciplines and the collaboration between countries. In recent years, due to the growing interest on new treatment strategies such as the theranostics personalized approach, the research has been centered on the production and application of emergent theranostic radionuclides, such as Cu-67, Sc-47. Alpha emitters, in particular Ra-223 and Ac-225, are also gaining attention, in particular in USA and Germany, and among conventional radionuclides, research on Lu-177 is constantly growing.

## Figures and Tables

**Table 1:** Relevant physical characteristics of principal conventional therapeutic radionuclides [17,18,19].

Radionuclide	Half-Life	E Max β^−^ (keV)	Production Method
^89^Sr	50.5 d	1496	^88^Sr(n,γ)^89^Sr
			^89^Y(n,p)^89^Sr
^90^Y	64.1 h	2280.1	^89^Y(n,γ)^90^Y
			^90^Sr/^90^Y gen.
^117m^Sn	13.6 d	130	^116^Sn(n,γ)^117m^Sn
		150	^117^Sn(n,n’,γ)^17m^Sn
			^116^Cd(α,3n)^117m^Sn
^131^I	8.02 d	606	^130^Te(n,γ)^131m,g^Te➔^131^I
^153^Sm	46.5 h	808.2	^152^Sm(n,γ)^153^Sm
^169^Er	9.40 d	350	^165^Ho(p,n)^165^Er
^177^Lu	6.73 d	497.8	^176^Yb(n, γ,β^−^)^177^Lu
			^176^Lu(n,γ)^177^Lu
^186^Re	3.72 d	1069.5	^185^Re(n,γ)^186^Re
			^186^W(p,n) ^186^Re
^188^Re	17.0 h	2120.4	^188^W/^188^Re gen.

**Table 2:** Relevant physical characteristics of principal emergent therapeutic radionuclides [8].

Radionuclide	Half-Life	Energy (keV)	Production Methods
^67^Cu	2.58 d	E_β_^−^_mean_ = 141 E_γ_ = 91; 93; 185	^68^Zn(p,2p)^67^Cu ^68^Zn(γ,p)^67^Cu ^70^Zn(p,α)^67^Cu ^70^Zn(d,αn)^67^Cu
^47^Sc	3.35 d	E_β_^−^_mean_ = 162 E_γ_ = 159	^47^Ti(n,p)^47^Sc ^46^Ca(n,γ)^47^Ca → ^47^Sc ^48^Ca(γ,n)^47^Ca → ^47^Sc
^166^Ho	26.82 h	E_β_^−^_mean_ = 665 E_γ_ = 81	^165^Ho(n,γ)^166^Ho ^164^Dy(2n,γ)^166^Dy → ^166^Ho
^161^Tb	6.89 d	E_β_^−^_mean_ = 154 E_γ_ =49; 75	^160^Gd(n,γ)^161^Gd → ^161^Tb
^212^Pb (^212^Bi)	10.64 h (60.6 min)	E_β_^−^_mean_ = 100 E_γ_ = 238; 300 (E_α_ = 6050; 6089)	^224^Ra/^212^Pb generator
^225^Ac	10.0 d	E_α_ = From 5637 to 5830 E_γ_ =99.8	^232^Th(p,2p6n)^225^Ac ^226^Ra(p,2n)^225^Ac ^226^Ra(γ,n)^225^Ra → ^225^Ac
^213^Bi	45.6 min	E_α_ = 5558; 5875 E_γ_ = 324	^225^Ac/^213^Bi generator
^223^Ra	11.4d	E_α_ = From 5433 to 5871 E_γ_ = 144; 154; 269; 324; 338	^227^Ac/^227^Th/^223^Ra gen.

**Table 3:** Theranostic radioisotopes pair [31].

Imaging/Therapeutic Pair	T_1/2_	Mode of Decay
Sc-44/Sc-47	3.9 h / 3.35 d	β^+^; EC; γ/β^−^; γ
Cu-64/Cu-67	12.7 h / 2.58 d	EC; β^−^; β^+^/β^−^; γ
Sr-83/Sr-89	32.4 h / 50.5 d	EC; β^+^; γ/β^−^
Ga-68/Ga-67	68 min / 3.26 d	β^+^/Aug; EC
Y-86/Y-90	14.7 h / 2.7 d	EC; β^+^; γ/β^−^
I-124/I-131	4.2 d / 8.0 d	EC; β^+^; γ/β^−^; γ
Tb-152/Tb-161	17.5 h / 6.9 d	EC; β^+^; γ/β^−^; γ
Tb-152/Tb-149	17.5 h / 4.1 h	EC; β^+^; γ/EC; α; β^+^

β^+^ = positrons; β^−^ = beta electrons; C.E. = conversion electrons; Aug. = Auger electrons.

**Table 4:** Theranostic radionuclides [19].

Radionuclide	T_1/2_ (days)	Principal E_γ_ for Imaging, keV (%)	Therapeutic Particle(s)
Sc-47	3.35	159 (68)	β^−^
Cu-67	2.58	186 (40)	β^−^
Ga-67	3.26	93, 184, 296, (40, 24, 22)	Aug., C.E.
In-111	2.8	171, 245, (91, 94)	Aug., C.E.
Sn-117m	14	159 (86)	C.E.
I-123	13.3 h	159 (83)	Aug., C.E.
I-131	8	365 (82)	β^−^
Sm-153	1.94	103 (30)	β^−^
At-211	7.2 h	79 (21)	α
Bi-213	46 min	441 (926)	β^−^; α (from TI-209 → Bi-213 decay chain)

β^−^ = beta electrons; C.E.= conversion electrons; α = alpha particles; Aug. = Auger electrons.

**Table 5:** Radionuclide data representing the number of papers extrapolated from the Scopus and Web of Science (WoS) databases published for each radionuclide over the last 10 years (2008–2018). (**a**) Conventional (CRN) vs. (**b**) emergent (ERN).

a			b		
CRN	Scopus	WoS	ERN	Scopus	WoS
^131^I	4458	3764	^67^Cu	102	119
^90^Y	1944	2305	^47^Sc	65	63
^177^Lu	1789	1192	^223^Ra	1115	642
^188^Re	596	411	^166^Ho	160	137
^186^Re	362	181	^161^Tb	28	181
^153^Sm	626	313	^149^Tb	21	10
^89^Sr	528	255	^212^Pb/^212^Bi	14	41
^186^Er	36	14	^225^Ac	231	145
Total	10339	8435	^213^Bi	252	141
			^211^At	75	12
			^117m^Sn	315	537
			Total	2378	1870

**Table 6:** Annual publications discussing CRNs (2008–2018).

Topic	2008	2009	2010	2011	2012	2013	2014	2015	2016	2017	2018	TOT
^131^I	491	429	445	448	475	466	399	404	330	342	229	4458
^90^Y	161	163	155	197	194	168	208	197	193	179	129	1944
^177^Lu	97	95	89	135	167	144	157	188	244	263	210	1789
^188^Re	94	75	58	61	54	53	50	42	46	40	23	596
^186^Re	50	40	42	36	44	43	18	23	25	29	12	362
^153^Sm	60	70	54	66	69	78	55	63	48	37	26	626
^89^Sr	47	54	47	43	77	71	42	41	38	47	21	528
^186^Er	5	6	1	6	4	2	6	2	1	0	3	36
Total	1005	932	891	992	1084	1025	935	960	925	937	953	10339

**Table 7:** Annual publications discussing ERNs (2008–2018).

Topic	2008	2009	2010	2011	2012	2013	2014	2015	2016	2017	2018	TOT
^67^Cu	10	11	7	8	12	5	13	12	6	11	7	102
^47^Sc	3	5	3	8	8	5	4	9	6	12	2	65
^223^Ra	27	9	22	3	108	142	159	164	152	165	134	1115
^166^Ho	15	16	12	16	15	21	13	18	13	8	13	160
^161^Tb	0	0	0	1	1	5	6	5	6	2	2	28
^149^Tb	1	0	0	5	1	1	4	1	3	3	2	21
^212^Pb/^212^Bi	1	0	0	0	4	1	3	0	1	3	1	14
^225^Ac	12	9	11	19	23	17	26	18	20	38	38	231
^213^Bi	28	22	16	27	22	30	25	21	15	26	20	252
^211^At	9	5	3	10	6	8	5	11	3	8	7	75
^117m^Sn	28	16	29	26	30	30	43	28	38	25	22	315
Total	134	93	103	153	230	265	301	287	263	301	248	2378

**Table 8:** Analysis of the popularity of different conventional radionuclides by the top 20 countries with the highest number of relevant publications (2008–2018). Highlighted in grey are the two highest percentages for each conventional radionuclide.

Rank	Country	CRN (Contribution % per country)							
		^131^I	^90^Y	^177^Lu	^188^Re	^186^Re	^153^Sm	^89^Sr	^186^Er
1	USA	22.21	24.72	17.99	18.25	15.66	14.68	21.73	6.52
2	Germany	5.88	9.86	14.63	9.05	9.39	5.73	5.73	23.91
3	China	10.22	3.85	1.79	11.70	2.92	3.67	4.99	0.00
4	Italy	5.01	7.65	7.51	5.15	6.52	6.46	4.11	6.52
5	United Kingdom	3.83	3.85	3.93	3.06	5.64	5.14	8.08	2.17
6	France	4.18	4.13	3.54	4.18	4.35	4.41	3.08	4.35
7	Nederland	2.91	3.61	7.34	2.79	5.22	2.64	2.50	4.35
8	Japan	5.47	2.04	0.79	1.11	3.13	1.76	8.32	0.00
9	India	2.87	2.52	0.00	5.29	2.71	4.55	4.85	17.39
10	Switzerland	1.40	2.88	4.41	1.81	2.09	1.91	1.32	0.00
11	Canada	1.81	1.76	3.28	2.65	1.67	2.50	3.08	0.00
12	Australia	1.47	2.68	3.32	1.25	2.71	1.76	1.17	0.00
13	Turkey	2.74	1.92	1.22	0.84	2.92	1.32	1.17	2.17
14	South Korea	3.02	1.40	1.31	2.92	0.84	0.44	0.44	0.00
15	Iran	1.51	1.32	2.31	2.51	2.30	7.49	1.32	0.00
16	Spain	1.86	2.80	1.00	0.00	4.38	1.91	2.06	4.35
17	Poland	1.86	2.36	1.83	1.67	1.46	2.06	0.73	2.17
18	Sweden	1.05	1.52	4.93	0.97	1.46	0.59	1.17	2.17
19	Belgium	1.46	2.12	1.62	1.67	2.30	1.47	1.47	2.17
20	Brazil	1.58	1.28	1.22	0.70	0.63	3.67	0.15	0.00

**Table 9:** Analysis of the popularity of different emergent radionuclides by the top 20 countries with the highest number of relevant publications (2008–2018). Highlighted in grey are the two highest percentages for each emergent radionuclide.

Rank	Country	ERN (Contribution % per Country)										
		^67^Cu	^47^Sc	^223^Ra	^166^Ho	^161^Tb	^149^Tb	^212^Pb/^212^Bi	^225^Ac	^213^Bi	^211^At	^117m^Sn
1	USA	13.48	13.25	28.73	20.73	3,85	19.35	21.05	35.94	31.97	30	13.99
2	Germany	9.22	4.82	8.09	1.22	3.85	9.68	5.26	17.19	22.40	5.45	3.96
3	United Kingdom	1.42	2.41	8.57	3.05	3.85	3.23	10.53	2.81	2.19	3.64	1.86
4	France	6.38	16.87	4.71	4.88	17.31	9.68	10.53	3.44	5.74	7.27	3.03
5	China	4.26	1.20	1.63	1.83	0.00	0.00	5.26	1.88	1.37	3.64	18.65
6	Italy	2.84	1.20	4.71	2.44	0.00	0.00	0.00	2.19	1.37	0.91	2.56
7	Japan	8.51	6.02	2.05	0.00	1.92	3,23	0.00	1.56	1.09	5.45	7.93
8	Sweden	1.42	1.20	2.35	1.83	5.77	3.23	5.26	1.88	3.83	21.82	0.93
9	Spain	0.71	0.00	4.59	0.61	0.00	0.00	5.26	0.94	0.55	0.00	1.63
10	Netherland	1.42	0.00	1.87	18.90	0.00	0.00	0.00	2.50	3.01	0.00	1.63
11	Australia	2.13	0.00	2.41	1.22	0.00	3.23	0.00	4.38	6.56	0.00	0.47
12	Canada	2.13	2.41	2.96	0.61	0.00	0.00	0.00	3.75	0.82	2.73	2.80
13	Switzerland	3.55	4.82	2.60	1.83	17.31	16.13	5.26	0.94	1.91	0.00	0.93
14	Russian Federation	5.67	2.41	1.27	2.44	11.54	9.68	0.00	4.69	2.19	0.91	1.63
15	India	1.42	3.61	0.84	5.49	1.92	6.45	5.26	1.25	1.64	0.00	5.83
16	South Korea	4.96	1.20	0.72	4.27	0.00	3.23	0.00	0.63	0.00	0.00	6.69
17	Belgium	0.71	0.00	1.57	3.05	9.62	0.00	5.26	1.56	0.82	0.91	0.93
18	Brazil	0.71	0.00	1.39	6.10	1.92	3.23	0.00	1.25	0.82	1.82	0.70
19	Norway	0.00	0.00	2.17	0.61	0.00	0.00	0.00	0.31	0.55	0.00	0.47
20	Austria	0.71	0.00	1.45	0.61	0.00	0.00	0.00	1.25	1.64	0.00	0.93

**Table 10:** Comparison of total publications, affiliation number for each radionuclide, and category of radionuclides.

**CRN**	**^131^I**		**^90^Y**		**^177^Lu**			**^188^Re**		**^186^Re**		**^153^Sm**			**^89^Sr**		**^186^Er**		**Total**
Papers	4458		1944		1789			596		362		626			528		36		10339
Affiliations	5429		2496		2290			718		479		681			681		46		12820
**ERN**	**^67^Cu**	**^47^Sc**		**^223^Ra**		**^166^Ho**	**^161^Tb**		**^149^Tb**		**^212^Pb/^212^Bi**		**^225^Ac**	**^213^Bi**		**^211^At**		**^117m^Sn**	**Total**
Papers	102	65		1115		160	28		21		14		231	252		75		315	2378
Affiliations	141	83		1657		164	52		31		19		320	366		110		429	3372

**Table 11:** Classification of Journal Subject Areas (JSAs) into macro-areas.

Macro-areas	JSA	Topic
Physics	Energy, engineering, physics and astronomy, materials science	Research on the production of radionuclides
Chemistry/Biology/Pharmacology	Agricultural and biological sciences, biochemistry, genetics and molecular biology, chemical engineering, chemistry, environmental science, pharmacology, toxicology, and pharmaceutics	Preclinical research, with particular reference to the development and in vitro/in vivo biological evaluation of radiopharmaceuticals
Medicine	Medicine	Clinical research
