# Correction: Uccelli, L., et al. Therapeutic Radiometals: Worldwide Scientific Literature Trend Analysis (2008–2018). *Molecules* 2019, *24*, 640

**DOI:** 10.3390/molecules24071308

**Published:** 2019-04-03

**Authors:** Licia Uccelli, Petra Martini, Corrado Cittanti, Aldo Carnevale, Loretta Missiroli, Melchiore Giganti, Mirco Bartolomei, Alessandra Boschi

**Affiliations:** 1 Department of Morphology, Surgery and Experimental Medicine, University of Ferrara, Via Ludovico Ariosto, 35-44121 Ferrara, Italy; licia.uccelli@unife.it (L.U.); corrado.cittanti@unife.it (C.C); aldo.carnevale@unife.it (A.C.); melchiore.giganti@unife.it (M.G.); alessandra.boschi@unife.it (A.B.); 2 Nuclear Medicine Unit, University Hospital, Via Aldo Moro, 8-44124 Ferrara, Italy; m.bartolomei@ospfe.it; 3 Legnaro National Laboratories, Italian National Institute for Nuclear Physics (LNL-INFN), Viale dell’Università, 2, 35020 Legnaro (PD), Italy; 4 Radiology University Unit, University Hospital, Via Aldo Moro, 8-44124 Ferrara, Italy; 5 Bibliometric and Databases Unit, Research Office, University of Ferrara, Via Ludovico Ariosto, 35-44121 Ferrara, Italy; loretta.missiroli@unife.it

The authors wish to make the following corrections to their paper [1]:

“Er-186” and “Erbium-186” should be corrected to “Er-169” and “Erbium-169”. In the Introduction section, on the eighth line of page 2 (Table 1), “17mSn” should be corrected to “117mSn”, and on the 12th line of page 2 (Table 1), “165Ho(p,n)^165^Er” should be corrected to “^168^Er(n,γ)^169^Er”.

The changes do not affect the scientific results. The authors would like to apologize for any inconvenience caused to the readers by these changes.
